# Designing an AI-Enhanced Public Health Care Platform for the Rapidly Aging Population in South Korea: Protocol for a Mixed Methods Study Based on the Design Thinking Approach

**DOI:** 10.2196/63094

**Published:** 2025-08-01

**Authors:** Jeongone Seo

**Affiliations:** 1 School of Communication & Information Studies, Rutgers University, 4 Huntington Street, New Brunswick, NJ, 08901, United States, 1 2013645142; 2 Research Center for Social Welfare, Sungkyunkwan University, Seoul, Republic of Korea

**Keywords:** aging society, design thinking, digital literacy, digital health care platform, participatory research, digital inclusion, older adults, user-centered design

## Abstract

**Background:**

South Korea is undergoing one of the world’s fastest demographic shifts toward an aging society, with projections indicating that by 2047, half of all households will be led by older adults. While digital health technologies such as mobile health apps and telemedicine offer promising solutions for promoting healthy aging and reducing health care expenditures, their adoption among older Koreans remains limited. To address these challenges, this study will use a user-centered design thinking approach to develop a public health care platform tailored to the needs of South Korea’s older adults.

**Objective:**

The primary objective of this study is to develop and evaluate a user-centered digital health care platform tailored to older adults in South Korea, with the aim of overcoming key barriers, such as low digital literacy, interface complexity, and mistrust in artificial intelligence–driven systems, and ultimately bridging the digital divide in health.

**Methods:**

This mixed methods study will integrate qualitative and quantitative research within a design thinking framework, progressing through 3 operational phases: empathize and define, ideate, and prototype and test. In phase 1, a scoping literature review, field observations at 5 community centers for older people and in-depth interviews with 29 older adults and 15 stakeholders were conducted to identify behavioral barriers and user needs. In phase 2, an open idea contest and expert focus groups were used to generate and prioritize innovative features for the platform. Phase 3 involves co-design workshops, minimum viable product development using a no-code platform, and usability testing with 8 to 10 older adults.

**Results:**

The research received a grant (HI22C1477) from the Korea Health Industry Development Institute (2023-2025) and ethical approval (2023-01-014) from Sungkyunkwan University. As of July 2025, data collection for phase 2 is ongoing, and preliminary findings are expected by late 2025.

**Conclusions:**

By adopting an inclusive design thinking approach, this study aims to produce a practical and user-centered platform for older adults. The findings will contribute to research on digital health equity and offer actionable insights for community-centered technology design.

## Introduction 

South Korea is experiencing one of the most rapid demographic shifts toward an aging population in the world, with official statistics indicating a steadily increasing proportion of older adults [1,2]. In 2020, the number of single-person households headed by older adults surpassed 1.66 million, constituting more than one-third of all such households [1]. Projections suggest that by 2047, half of all Korean households will be headed by an older adult [2]. While this demographic change reflects advances in health care and socioeconomic development, it also raises urgent questions about maintaining older adults’ health, well-being, and quality of life [3]. Many older Koreans live with multiple chronic conditions and face substantial health care costs, and approximately 9.5% require long-term care [1]. In response, the government has emphasized policy-level interventions and the development of innovative service models for healthy aging [2,3]. Among these, digital health technologies, such as mobile health apps, telemedicine, and remote monitoring, are considered promising strategies for reducing the burden on the health care system [2]. However, actual adoption among older adults remains low, hindered by barriers such as limited awareness, low digital literacy, and skepticism toward technology-driven interventions [4-6].

This discrepancy underscores a broader “digital divide” in health—a gap in access to, and effective use of, digital resources that often correlates with age, socioeconomic status, and technological proficiency [7-9]. Digital literacy plays a pivotal role in bridging this divide, encompassing not only the ability to operate devices but also to critically evaluate and apply digital information [4,7,9]. While younger cohorts in South Korea frequently acquire digital competencies through daily life and formal education, older adults have had fewer opportunities to develop such skills [6,10]. Although many own smartphones, use is often limited to basic communication rather than health management tasks [5]. Additional hurdles include text-heavy interfaces, foreign-language prompts, and frequent software updates that can further deter sustained use [11,12].

Against this backdrop, design thinking offers a user-centered, iterative approach to creating digital health solutions that genuinely reflect the needs and preferences of older adults [13-15]. Characterized by 5 key stages—empathize, define, ideate, prototype, and test—design thinking actively engages end users, reframes problems from their perspectives, and iterates solutions based on real-world feedback [13,14]. In this study, we group these 5 stages into 4 operational phases to streamline the design process: (1) emphathize and define, (2) ideate, and (3) prototype and test. Prior studies have shown that co-designing technology with older adults not only increases adoption but also fosters a sense of ownership and comfort in using platforms that aligns with their cognitive and physical capabilities [6,16,17]. These findings suggest that participatory, empathy-driven methodologies can narrow the digital divide by identifying context-specific user requirements early in the design process [16,18].

In this study, we use a design thinking framework to develop a public health care platform tailored to older adults in South Korea. Our goal is to create a mobile- or web-based platform that connects older adults to essential health information and services, such as chronic disease management, medication reminders, and social welfare resources, in a user-friendly and contextually relevant manner. We hypothesize that a co-designed, user-centric platform will demonstrate enhanced usability and acceptance, potentially leading to better health management behaviors and reduced health care costs [2,3]. By integrating older adults throughout the design process, we aim to address their unique barriers, mitigate the digital divide, and ultimately contribute to a more inclusive health care system.

## Methods

### Overview

This study uses a participatory, mixed methods approach grounded in design thinking [13-15] to create a user-centered digital public health care platform for older adults in South Korea. We apply the 5 classical stages of design thinking—empathize, define, ideate, prototype, and test—grouped into 3 operational phases over a 36-month period (January 2023 to December 2025). While the design thinking process is structured into phases, these phases are not implemented in a strictly linear order. Insights from earlier activities are continuously cycled back into later stages, allowing for iterative refinement across the project timeline.

**Figure 1.**
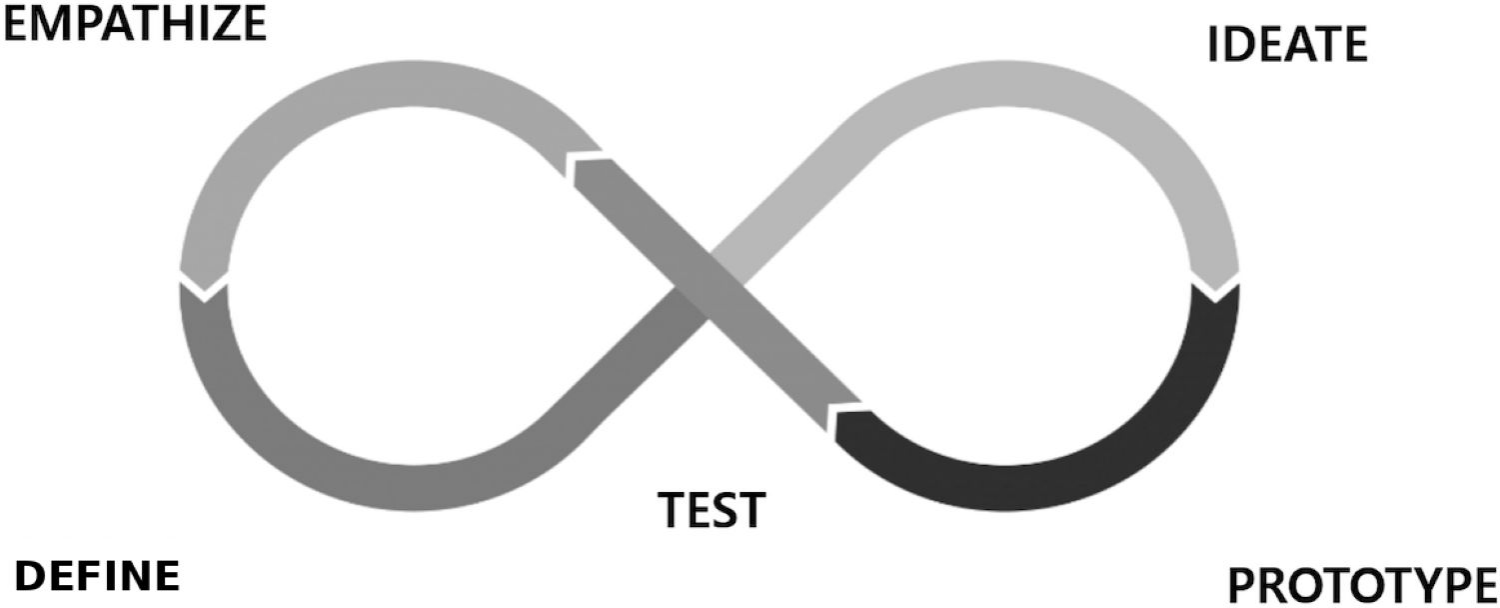
Five key stages of the design thinking process.

Although this study is organized into 3 distinct operational phases, the activities within these phases are conducted iteratively and concurrently rather than strictly sequentially, reflecting the inherent flexibility and iterative nature of the design thinking methodology [13,14,16]. Thus, these phases permit ongoing feedback loops and dynamic adjustments based on user insights and emergent findings. Figure 1 provides a schematic diagram illustrating how qualitative (eg, interviews and observations) and quantitative (eg, System Usability Scale; SUS) data converge to refine the platform’s design.

The design thinking process adopted in this study consists of 5 iterative stages: empathize, define, ideate, prototype, and test [13-15]. *Empathize* involves understanding users’ needs, preferences, and behaviors through methods such as interviews, observations, and field immersion. This helps uncover deeper insights that inform problem framing [13,14]. *Define* focuses on synthesizing user insights to identify key challenges and articulate a clear problem statement. A well-defined problem sets the direction for ideation and solution development [13]. *Ideate* encourages the generation of diverse ideas in response to the defined problem, often through creative techniques such as brainstorming and mind mapping [13]. *Prototype* refers to building tangible representations of promising ideas. These prototypes help visualize concepts and gather early feedback before full development [13,15]. *Test* involves evaluating prototypes with real users to identify usability issues, gather insights, and refine solutions iteratively. This stage ensures the solution is feasible, desirable, and aligned with users’ needs [14,15]. Together, these stages provide a user-centered and flexible framework that will guide the platform development process in this study.

To facilitate transparency in the mixed methods design, Table 1 summarizes how each design thinking stage integrates qualitative and quantitative insights [13,16,19] and Table 2 shows the mixed methods flow across the design thinking stages.

**Table 1.** Overview of study phases, activities, and outputs.

Phase	Timeline	Primary activities	Outputs
Phase 1: empathize and define	Jan 2023 to May 2025	Scoping review, field observation, semistructured interviews	Personas, scenario maps, problem statements
Phase 2: Ideation	Jan to Mar 2023 (students), fall 2025 (experts)	Idea contest, expert focus group discussions	Ranked ideas, priority features, technical feasibility roadmap
Phase 3: prototype and test	Jun to Dec 2025	Co-design workshops, minimum viable product development, usability testing	Functional prototype, usability metrics, user feedback

**Table 2.** Mixed methods flow across design thinking phases.

Design thinking stage	Input (qualitative and quantitative)	Process	Output
Phase 1: empathize and define	Scoping review (qualitative), interviews (qualitative), field notes (qualitative)	Persona creation, scenario mapping	Problem statements, user archetypes
Phase 2: ideate	Idea contest entries (qualitative), expert focus group discussions (qualitative)	Thematic coding, feasibility scoring	Ranked feature sets, concept clustering
Phase 3: prototype and test	Usability survey (quantitative: System Usability Scale), posttest interviews (qualitative)	Triangulation, sprint revision	Usability dashboard, minimum viable product refinement plans

This structure aligns with the mixed methods approach of Creswell and Plano Clark [19], ensuring both methodological rigor and iterative design adaptability.

### Phase 1: Empathize and Define Scoping Literature Review

#### Overview

A scoping review was conducted in accordance with the PRISMA-ScR ( Preferred Reporting Items for Systematic Reviews and Meta-Analyses Extension for Scoping Reviews) guidelines [20] and the Arksey and O’Malley [21] framework to investigate how older adults interact with digital healthcare platforms, focusing on human information behavior and associated barriers. Four major academic databases (PubMed, Scopus, Web of Science, and gray literature repositories) were searched using Boolean combinations of “eHealth,” “digital literacy,” “elderly,” and “technology adoption,” covering the period from 2010 to 2023. Two independent reviewers screened titles and abstracts and then the full texts. Data extracted included study design, population characteristics, technology type, behavioral frameworks, and outcome measures. Previous studies indicate that older adults’ acceptance of digital health tools can be influenced by factors such as interface complexity and perceived ease of use [22,23]. From this review, we identified 4 prominent behavioral barriers: interface complexity, low digital literacy, emotional resistance, and lack of contextual support [5,6,11]. These findings informed the design of subsequent interview protocols, field observation criteria, and participant personas. They also shaped the problem statements and discussion prompts used during the ideation phase (contest and expert focus groups).

#### In-Depth Interviews: Participants and Recruitment

We aimed to recruit approximately 29 older adult participants (aged 65 years or older) from Seoul and Suwon, balancing factors like digital experience level and living situations to ensure thematic saturation (Textbox 1). Previous gerontological research suggests that samples of around 20 to 30 participants can capture major themes while allowing demographic variability [24].

Textbox 1. **Inclusion and exclusion criteria.**

**Inclusion criteria**

Korean-speaking, cognitively able to participate, with or without prior smartphone experience

**Exclusion criteria**

Diagnosed dementia or psychiatric disorders impeding informed consent or interview participation

Stakeholders included social workers, care coordinators, nurses, and program administrators, each with at least 1 year of experience delivering services to older adults. The sample size (n=15) aimed to represent the range of professional roles relevant to care of older people and welfare service systems [3]. Recruitment occurred through program referrals at community centers for older people and snowball sampling. Interested individuals received a study information sheet and provided signed informed consent [6].

#### In-Depth Interviews: Interview Procedure

Interviews were conducted in Korean, typically lasting 40 to 90 minutes. Depending on participant preference and mobility, interviews were held face-to-face or by phone from September 2024 to May 2025. A semistructured guide (Multimedia Appendix 1) covered topics such as older adults’ digital device use, barriers, support needs, safety concerns, and impacts on quality of life. Interviews were audio-recorded, transcribed verbatim, deidentified, and uploaded to a secure server.

#### In-Depth Interviews: Data Analysis

**Figure 2.**
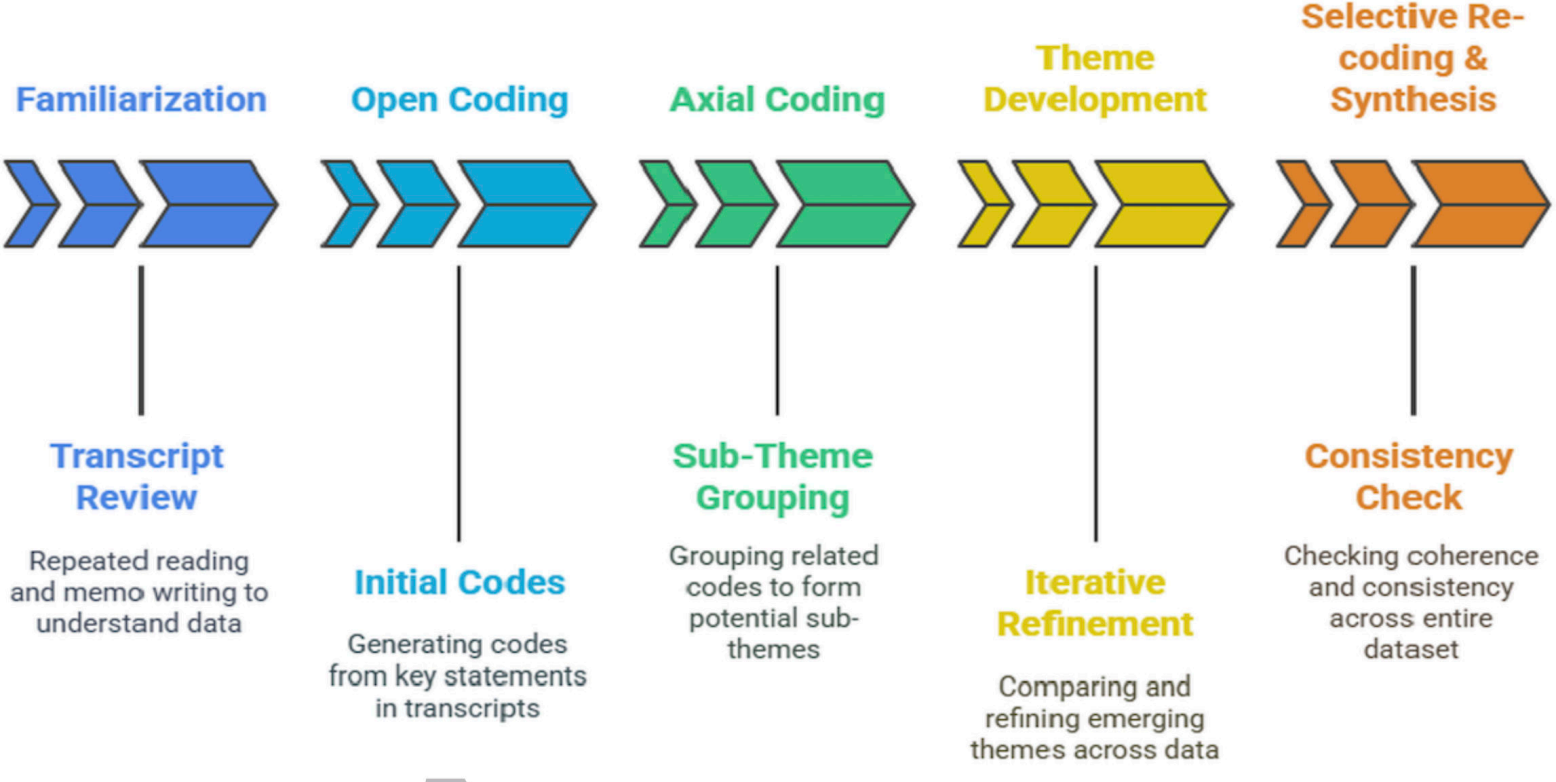
Data analysis process using reflexive thematic analysis

Data were analyzed using reflexive thematic analysis [25]. The process is outlined in Figure 2.

Coding was facilitated by NVivo (version 14; Lumivero). To enhance the credibility of findings, the research team used member-checking with selected participants, peer debriefing sessions for code refinement, and triangulation with field observation data and the scoping review outcomes [16,25].

### Phase 2: Ideate

#### Student Idea Contest

In January 2023, we launched an open idea contest titled Social Welfare+Digital Technology to gather innovative solutions targeting older adult health care needs. Promotion was conducted via online university platforms (Everytime and Instagram). We enrolled students or recent graduates in social welfare–related fields. The submission requirements were a 5-page proposal outlining the problem background, a proposed digital solution, and an implementation plan. The review criteria were innovation (25% weight), user-centeredness (25% weight), feasibility (20% weight), alignment with eldercare goals (20% weight), and clarity/scalability (10% weight). The review criteria and weights were determined by the research team based on relevance to digital eldercare innovation and user-centered design principles.

We received 24 proposals, and 6 won awards based on feedback from an expert panel (including a social worker, physician, and technology entrepreneur). Notable ideas, such as a voice-based health assistant, were prioritized for prototype development [15,16].

#### Expert Focus Group

Scheduled for late 2025, focus groups will involve 8 to 10 professionals with expertise in user experience and user interface design, geriatrics, and public health. This range (n=8 to 10) was chosen to ensure multidisciplinarity while maintaining an optimal group size for in-depth discussions [26]. Using the personas and problem statements derived from phase 1, participants will evaluate the feasibility of the idea contest winners, prioritize key features and components (via dot-voting and feature scoring), and discuss integration paths with existing public systems (eg, the National Health Insurance Service). Outputs from this stage will inform a minimum viable product (MVP) blueprint for development [15,16].

### Phase 3: Prototype and Test

#### Co-Design and Development

Between June and September 2025, we will adopt an agile co-design process. Two older adult advisors will collaborate with user experience designers, front-end developers, and back-end engineers to create a functional MVP. Low- to medium-fidelity wireframes will be generated using Figma, and the MVP will be implemented with Bubble.io (a no-code platform) for rapid iteration [16]. Initial core features include simplified login and intuitive navigation, medication reminders with optional voice prompts, and an “ask a nurse” chatbot interface for basic health queries.

#### Usability Testing

A subset of 8 to 10 older adult users from the interview cohort will be recruited for usability testing, following the heuristic of Nielsen and Landauer [27] that testing with 5 to 10 users can uncover 80% to 90% of major usability problems [5,23]. Testing will occur in October to November 2025 through scheduled on-site sessions (Textbox 2) . Participants will perform key tasks on the MVP, including logging into the app, setting or modifying medication reminders, and using the chatbot for health-related questions.

A think-aloud protocol will capture real-time user reactions, and screen recordings will log errors or confusion points.

Textbox 2. **Usability testing data collection and analysis.**

**System Usability Scale (SUS)**

After completing the tasks, participants will fill out the SUS questionnaire. Results will be analyzed via descriptive statistics (mean and SD) using SPSS (version 28; IBM Corp), with a score ≥68 considered “acceptable.”

**Posttest interview**

Participants will provide open-ended feedback on overall design, satisfaction, and suggested improvements. Thematic coding will be used to identify common pain points [25].

### Mixed Methods Integration

To ensure robust triangulation and iterative refinement, we integrate qualitative and quantitative data at 2 critical junctures [13,16,19] through interpretive convergence. Themes from phase 1 interviews and field observations are mapped against usability bottlenecks detected in phase 3 (SUS scores and error logs) to deepen our understanding of how older adults’ perceptions and attitudes translate into actual platform interaction challenges.

By weaving together data from multiple sources, this approach strengthens the ecological validity of our findings and ensures that the final platform design is both technically sound and responsive to real-world user contexts [13,16,19].

### Ethical Considerations

This study was approved by the Institutional Review Board of Sungkyunkwan University (2023-01-014). Prior to participation, all participants were provided with both written and verbal explanations of the study objectives, procedures, potential risks, and benefits. Written informed consent was obtained from every participant. To maintain confidentiality, no personal identifiers were collected; audio recordings and transcripts were stored on password-protected devices. All participants received KRW 30,000 (US $21.83) as compensation for their participation.

## Results

As of April 2025, phase 1 (empathize and define) had been completed, including 29 older adult interviews and 15 stakeholder interviews. These insights will directly inform phases 2 and 3, which are planned to be carried out in the latter half of 2025.

During the scoping review, we screened publications from 2010 to 2023 and identified key barriers to older adults’ engagement with digital health tools, including interface complexity, low digital literacy, emotional resistance, and insufficient contextual support. These categories informed the initial interview protocol and guided the creation of personas. Preliminary qualitative analysis suggests three recurring themes: (1) interface anxiety, (2) family-mediated use, and (3) trust in artificial intelligence (AI)–driven systems. First, participants reported anxiety when confronted with unfamiliar buttons, icons, or English terminology. They often deferred to family members or care workers to handle advanced features. Second, many older adults rely on adult children or relatives to set up smartphones, download health apps, and troubleshoot technical issues. This reliance creates both a support system and a dependency, potentially limiting independent engagement. Third, several older adults expressed skepticism about chatbot-based health advice, citing concerns over accuracy, privacy, and data misuse. Stakeholders stressed the importance of transparent data governance and easy-to-understand consent processes.

The observed difficulties and reported anxieties underscore the need for simplified interfaces. These insights will shape the concept brief for the co-design sessions, ensuring that user-friendly layouts and robust support features (eg, step-by-step tutorials) are prioritized. The findings on mistrust of AI will be crucial in determining the extent to which automated health recommendations are integrated into the MVP and how transparency in data handling is communicated. The research received a grant (HI22C1477) from the Korea Health Industry Development Institute (2023-2025) and ethical approval (2023-01-014) from Sungkyunkwan University. Recruitment progressed smoothly through community partnerships, though ongoing efforts are required to ensure a balanced representation of older adults with varying degrees of digital literacy.

## Discussion

### Principal Findings

This research protocol outlines a design thinking–based approach to address the ongoing challenge of low digital health care adoption among older adults in South Korea. By combining a scoping review, field observations, and in-depth interviews, the study seeks to uncover barriers such as interface anxiety and trust in AI-driven systems. These insights are expected to inform the design of a user-centered platform that is both inclusive and usable.

### Comparison to Prior Work

The proposed co-design process builds upon prior participatory approaches, but places a stronger emphasis on iterative feedback loops with older adult participants. While the participatory framework is anticipated to improve alignment with user needs, its actual impact will depend on successful implementation and engagement throughout the design phases.

### Strength and Limitations

A potential strength of this study lies in its planned integration of multiple qualitative and quantitative methods across iterative design stages. However, certain limitations, such as the initial focus on urban participants and a relatively short MVP evaluation period, may restrict early generalizability. These limitations will be addressed in subsequent phases by expanding to rural communities and implementing longer-term follow-up activities.

### Future Directions

Future work will explore scaling the platform to different demographic groups and health care contexts, including collaborations with local clinics and nonurban community centers. Usability evaluation in real-world settings will be prioritized to ensure long-term adoption.

### Dissemination Plan

Upon completion, the study aims to provide a transferable model of digital health engagement that can inform future interventions. Planned dissemination includes academic publishing, stakeholder workshops, and the development of publicly available co-design toolkits. Overall, this protocol contributes to growing efforts to bridge the digital divide by centering older adults as active agents in the design of inclusive health care technologies.

### Conclusions

This study will generate practical and theoretical insights into inclusive platform design by engaging older adults in a human-centered participatory process. The expected outcomes will inform digital health policy and community-based intervention models for aging societies.

## Supplementary material

10.2196/63094 Multimedia Appendix 1 Development of a smart care service platform model based on the Digital Literacy for Older Adults Interview Questionnaire.

## Abbreviations

AI: artificial intelligence
HCI: human-computer interaction
MVP: minimum viable product
PRISMA-ScR: Preferred Reporting Items for Systematic Reviews and Meta-Analyses Extension for Scoping Reviews
SPSS: Statistical Package for the Social Sciences
SUS: System Usability Scale
