# Supplementary material for: Designing an AI-Enhanced Public Health Care Platform for the Rapidly Aging Population in South Korea: Protocol for a Mixed Methods Study Based on the Design Thinking Approach
Source: JMIR Res Protoc. 2025 Aug 1;14:e63094. doi: 10.2196/63094 (PMC12360830; doi:10.2196/63094)
Supplement: Multimedia Appendix 1 [file resprot-v14-e63094-s001.docx]

## APPENDIX A

**Development of a Smart Care Service Platform Model Based on Digital Literacy for Older Adults** **Interview Questionnaire**

**Understanding Current Use of Digital Devices**

- Among the older adults you care for, what proportion uses digital devices?
- What are the main purposes or functions for which they use digital devices?
- Are there any common characteristics among those who use digital devices? (e.g., education level, economic status, personality traits, etc.)

**Barriers and Difficulties in Use**

- What are the main reasons older adults avoid using digital devices?
- What aspects of using digital devices do they find most difficult?
- When do they most often feel frustrated or experience failure when learning new technology?

**Support and Education**

- What is the most effective method to teach older adults how to use digital devices?
- Have you received any education or training on digital technology as a caregiver? If so, what did it cover?
- What kinds of support from institutions or the government would be helpful?

**Safety and Security**

- What safety or security concerns are most worrisome regarding digital device use?
- What kind of education or support is needed to prevent fraud such as voice phishing scams?
- What tips or guidelines do you give to older adults to protect their personal information?

**Future Prospects**

- How can technologies like AI and IoT be used in elder care?
- How do you think future generations of older adults will use digital devices 10 years from now?
- How do you expect digital technologies to impact the daily tasks of caregivers?

**Quality of Life Improvement**

- In what ways can the use of digital devices improve the quality of life for older adults?
- What impact does it have on maintaining social relationships or supporting emotional well-being?
- Can digital device use help older adults live more independently?

## APPENDIX B

Evaluation Criteria for the Digital + Social Welfare Idea Competition

| \| No. \| \| --- \|  \|  \| \| --- \|  \|  \| \| --- \|  \|  \| \| --- \| | Name of Lead Applicant | Necessity of Solving Social Problem (25%) | Feasibility of Technology Application (25%) | Methodological Appropriateness (25%) | Originality and Expected Research Impact (25%) |
| --- | --- | --- | --- | --- | --- | --- | --- | --- | --- |
|  |  |  |  |  |  |
|  |  |  |  |  |  |
|  |  |  |  |  |  |
|  |  |  |  |  |  |
|  |  |  |  |  |  |
